# Response to comment on 'Lack of evidence for associative learning in pea plants'

**DOI:** 10.7554/eLife.61689

**Published:** 2020-09-10

**Authors:** Kasey Markel

**Affiliations:** Department of Plant Biology, University of California, DavisDavisUnited States; Johns Hopkins UniversityUnited States; University of LausanneSwitzerland

**Keywords:** associative learning, pisum sativum, replication, phototropism, plant growth, Other

## Abstract

In 2016 Gagliano et al. reported evidence for associative learning in plants (Gagliano et al., 2016). A subsequent attempt to replicate this finding by the present author was not successful (Markel, 2020). Gagliano et al. attribute this lack of replication to differences in the experimental set-ups used in the original work and the replication attempt (Gagliano et al., 2020). Here, based on a comparison of the two set-ups, I argue that these differences are unable to explain the lack of replication in Markel, 2020.

## Introduction

If reproducible, the evidence for associative learning in plants reported in Gagliano et al., 2016 would require scientists to rethink the evolutionary history of learning because associative learning has only been consistently reported in metazoa. While learning does not imply phenomenological consciousness (Nagel, 1974), its possible discovery in plants nonetheless raises interesting questions about philosophy of mind (Bronfman et al., 2016), in addition to questions regarding the molecular mechanisms and evolutionary history of learning (Ginsburg and Jablonka, 2010).

The primary criticism of my replication attempt (Markel, 2020) is that light was not shown to be a reliable unconditioned stimulus (Gagliano et al., 2020). It is true that the plants in Markel, 2020 did not always grow towards the last presentation of light, which reduced the power to detect associative learning. However, Gagliano et al., 2016 does not emphasize how surprising it is that plants would always grow towards a one hour presentation of light preceded by presentations in the opposite direction. Most experiments with phototropism involve light exposure from only one side and for substantially longer periods of time (3 hr in Schumacher et al., 2018 and Haga and Kimura, 2019 3–6 hr in Sullivan et al., 2016 or 24 hr in Goyal et al., 2016). Relatively few experiments have been reported on phototropism in etiolated *Pisum sativum* seedlings. However, in wild-type etiolated *Arabidopsis thaliana* seedlings, phototropic bending is barely initiated after 1 hr of exposure (Sullivan et al., 2016). Moreover, circumnutation means that it is unlikely that phototropic bending would result in the growth of *Pisum sativum* into the predicted arm of the maze many hours later.

Furthermore, in phototropism experiments blue light illumination is generally presented horizontally to maximize the phototropic bending, but in the Y-maze configuration the angle of illumination comes primarily from above: if the LEDs are attached on the outside edges of the Y-maze, as in Markel, 2020, the angle of the light at soil level is less than 40° from vertical. If the lights are attached in the center of each arm of the Y-maze, the angle is less than 25° from vertical. This oblique light is unlikely to cause a phototropic response as rapid or as strong as that caused by horizontal light (see, for example, figure 1 in Sullivan et al., 2016). For technical reasons, neither Gagliano et al., 2016 nor Markel, 2020 measured the rate of phototropic bending.

Gagliano et al. mention that they also encountered conditions in which light was not an effective unconditioned stimulus, citing specifically their second experiment with various circadian phases. However, Markel, 2020 was performed in the same circadian phase as their first experiment, so circadian phase is not a candidate explanation for the less consistent phototropic growth.

**Figure 1.**
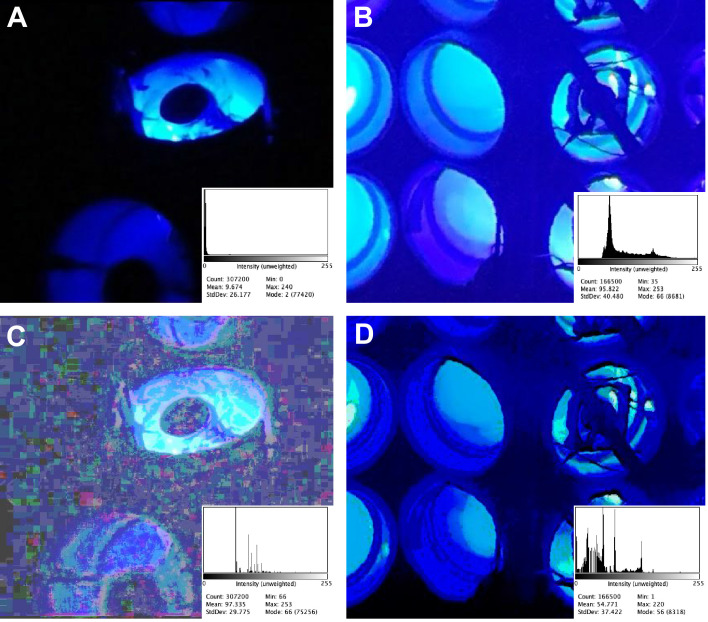
Comparison of background light levels in Gagliano et al., 2016 (left) and Markel, 2020 (right). The chambers in both experiments were lit only by blue lights within the Y-mazes; the only other light in the experiments came from the red headlamps worn by the experimenters. (**A**) Unedited still frame extracted from supplementary video 1 in Gagliano et al., 2016. (**B**) Unedited image of the Y-mazes used in Markel, 2020. (**C**) Panel A after histogram matching with panel B. The irregular rectangles are caused by the compression algorithm used in the original .mov file, and can be seen by adjusting the brightness and contrast on any frame extracted from that video. (**D**) Panel B after histogram matching with panel A. The histogram for each panel is shown in the bottom right corner; all histograms are 300 × 240 pixels, RGB, 281K; all images were processed as. png files for lossless compression. Image analysis was performed using the Fiji distribution of ImageJ Version 2.0.0-rc-69/1.52 n, Build: 269a0ad53f. The HistogramMatcher script is from the Fiji project CorrectBleach. This script was provided by Stack Overflow user Jan Eglinger, and is available in Supplementary file 1.

Gagliano et al. also mention that their plants were ~20 cm apart (a detail not included in the 2016 paper; moreover, in supplementary video 1 for this paper the spacing between the plants appears to be less than 20 cm, though greater than the compact spacing used in Markel, 2020). Regardless, the LED within each Y-maze provides the vast majority of the light to the seedlings, as can be seen from the internal illumination of the 'dark' arms of the Y-mazes in all panels of Figure 1. Moreover, because all the lights were on simultaneously, the higher level of background light in Markel, 2020 was only present when the larger amount of light from Y-maze internal reflection was also present.

I have used a technique called histogram matching (Chang and Wong, 1978) to compare images of the experimental chamber in Gagliano et al., 2016 (using a frame extracted from supplementary video 1; Figure 1A) and Markel, 2020 (using a photograph; Figure 1B). This technique adjusts the intensity histograms of images (the distribution of pixel intensity from 0 to 255 in the case of 8-bit images such as these) in a way that allows relative comparisons of brightness, contrast, and intensity to be made between images acquired with different settings. This technique is well suited to this particular case because there is a common standard of brightness (LEDs emitting 14 μmol m^−2^ s^−1^ at wavelengths between 430 and 505 nm). The unedited images in Figure 1A and B show a large apparent difference in background brightness. However, histogram-matched images in Figure 1C and D suggest that most of the apparent difference in brightness between the two studies is due to differences in settings and hardware of the cameras used to capture the images.

Nevertheless, the closer proximity of plants used in the replication attempt (Markel, 2020) resulted in a higher level of background light. The chambers used in the replication attempt were also smaller than those used in Gagliano et al., 2016, but all were maintained at the same temperature and humidity conditions. The difference in chamber size is unlikely to be important except insofar as it resulted in changes to plant spacing. Despite considerable effort to match the experimental details of the 2016 experiment, the replication attempt did not find evidence for associative learning in pea plants. Of course, this does not rule out the existence of such learning, and I sincerely hope that future research demonstrates the phenomenon to be reproducible.

## Data Availability

No new data was generated in this study; the methods to reproduce the analysis in Figure 1 are included in Supplementary file 1.

## Additional files

Supplementary file 1. Script used in the preparation of Figure 1.

Transparent reporting form
