# Supplementary material for: Response to comment on 'Lack of evidence for associative learning in pea plants'
Source: eLife. 2020 Sep 10;9:e61689. doi: 10.7554/eLife.61689 (PMC7556859; doi:10.7554/eLife.61689)
Supplement: Supplementary file 1. [file elife-61689-supp1.docx]

**Response to Comment on “Lack of evidence for associative learning in pea plants”**

**Supplemental file 1: Fiji script for histogram matching**

Kasey Markel^1^

^1^Department of plant biology, University of California, Davis, United States

import ij.IJ;

import histogram2.HistogramMatcher;

// get first image

imp1 = IJ.openImage("Image to be adjusted");

// get second image

imp2 = IJ.openImage("Image with histogram to serve as reference");

ip1 = imp1.getProcessor();

ip2 = imp2.getProcessor();

hist1 = ip1.getHistogram();

hist2 = ip2.getHistogram();

matcher = new HistogramMatcher();

newHist = matcher.matchHistograms(hist1, hist2);

ip1.applyTable(newHist);

imp1.setProcessor(ip1);

imp1.show();

imp2.show();

// show the histograms of both images

IJ.run(imp1, "Histogram", "");

IJ.run(imp2, "Histogram", "");

Image analysis was performed using the Fiji distribution of ImageJ Version 2.0.0-rc-69/1.52n, Build: 269a0ad53f. The HistogramMatcher script is from the Fiji project CorrectBleach. The script used was provided by Stack Overflow user Jan Eglinger, and is presented with minor modifications.
